# Sigmoid Adenocarcinoma Discovered in an Incarcerated Inguinal Hernia

**DOI:** 10.7759/cureus.89679

**Published:** 2025-08-09

**Authors:** Ioannis Katsarelas, Miltiadis Chandolias, Panagiotis Nachopoulos, Mohammad Hussamieh, Periklis Dimasis

**Affiliations:** 1 Department of Surgery, General Hospital of Katerini, Katerini, GRC

**Keywords:** acute surgical abdomen, hartmann’s operation, incarcerated inguinal hernia, mechanical large bowel obstruction, sigmoid adenocarcinoma, visceral surgery

## Abstract

Large bowel obstruction is a known surgical emergency. Common causes include malignancies of the colon and benign conditions, including hernias, adhesions, and other less common conditions such as inflammatory bowel disease, diverticular disease, and tuberculosis. However, the coexistence of hernias and colon neoplasm is a rare finding, one that is usually identified intraoperatively. In addition, there is no consensus on the best treatment route. We present a case of an 86-year-old male who presented in an emergency setting with an initial diagnosis of intestinal obstruction and an incarcerated left inguinal hernia. Upon exploratory laparotomy, the content was discovered to be an obstructed sigmoid colon due to a sigmoid colon neoplasm.

## Introduction

Colonic obstruction is a well-known medical emergency that will most likely require emergency intervention. Patients can present with minimal symptoms, all the way to severe dehydration, acute abdomen, and sepsis [1]. Large bowel obstruction etiologies are mostly mechanical, which include tumors, hernias, adhesions, inflammatory causes, and volvulus, but there are also functional causes like pseudo-obstruction of the colon (Ogilvie syndrome) and other rarer causes [2]. Possible symptoms include non-passage of feces and/or flatus, abdominal pain, distention, and absent bowel movements, many of which can vary, depending on the cause or anatomical site of the blockage [1].

## Case presentation

**Figure 1:**
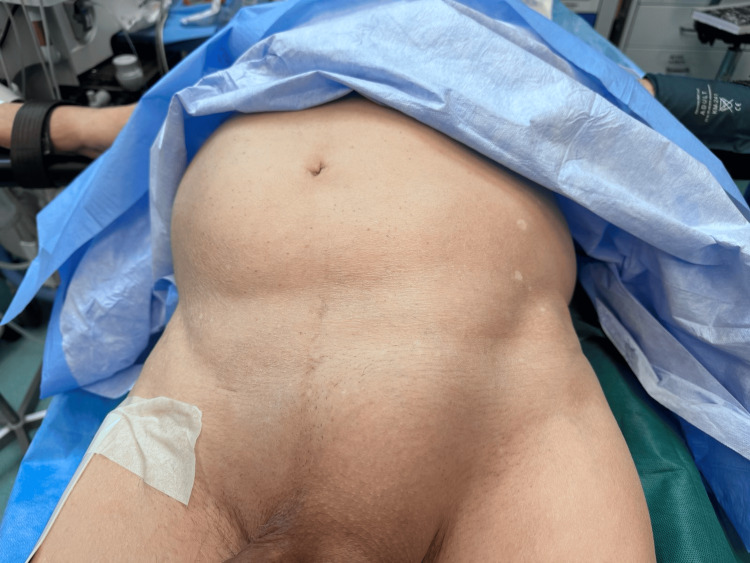
Left inguinal hernia accompanied by abdominal distention.

We present a case of an 86-year-old male patient who presented to the emergency department of our hospital, complaining about no defecation in the past week, accompanied by mild abdominal pain, which was greater in the last 24 hours, and feeling increasingly fatigued. On physical examination, abdominal distention was evident along with an irreducible left inguinal hernia, which was slightly tender, as was the rest of the abdomen (Figure 1). Auscultation revealed absent bowel sounds, and the digital rectal exam was negative for feces or blood.

**Figure 2:**
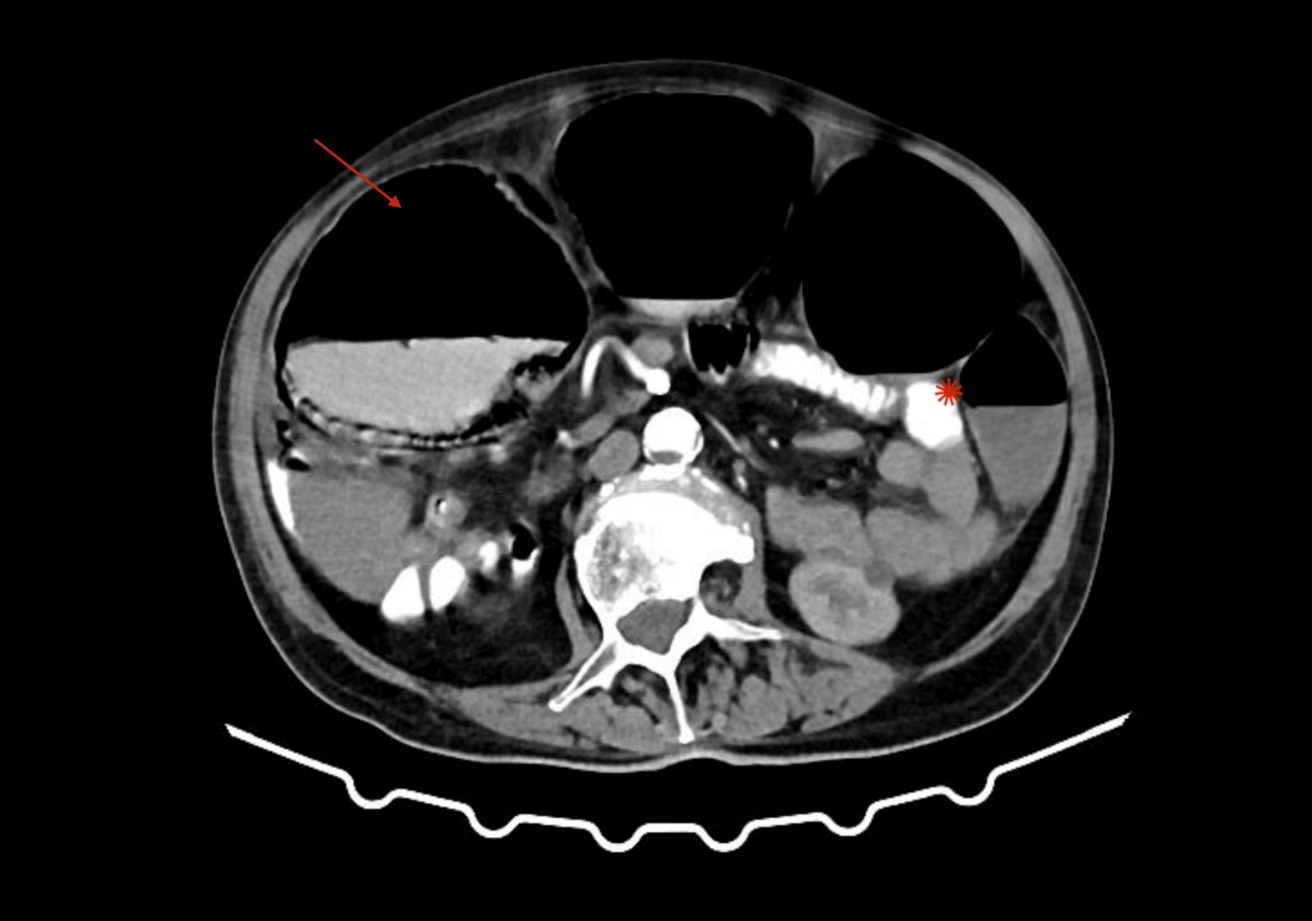
Computed tomography of the dilated large bowel (arrow), with normal small bowel (star). Large bowel obstruction with distention of the ascending and transverse colon is evident, while the small bowel is unaffected, evident of a competent ileocecal valve.

**Figure 3:**
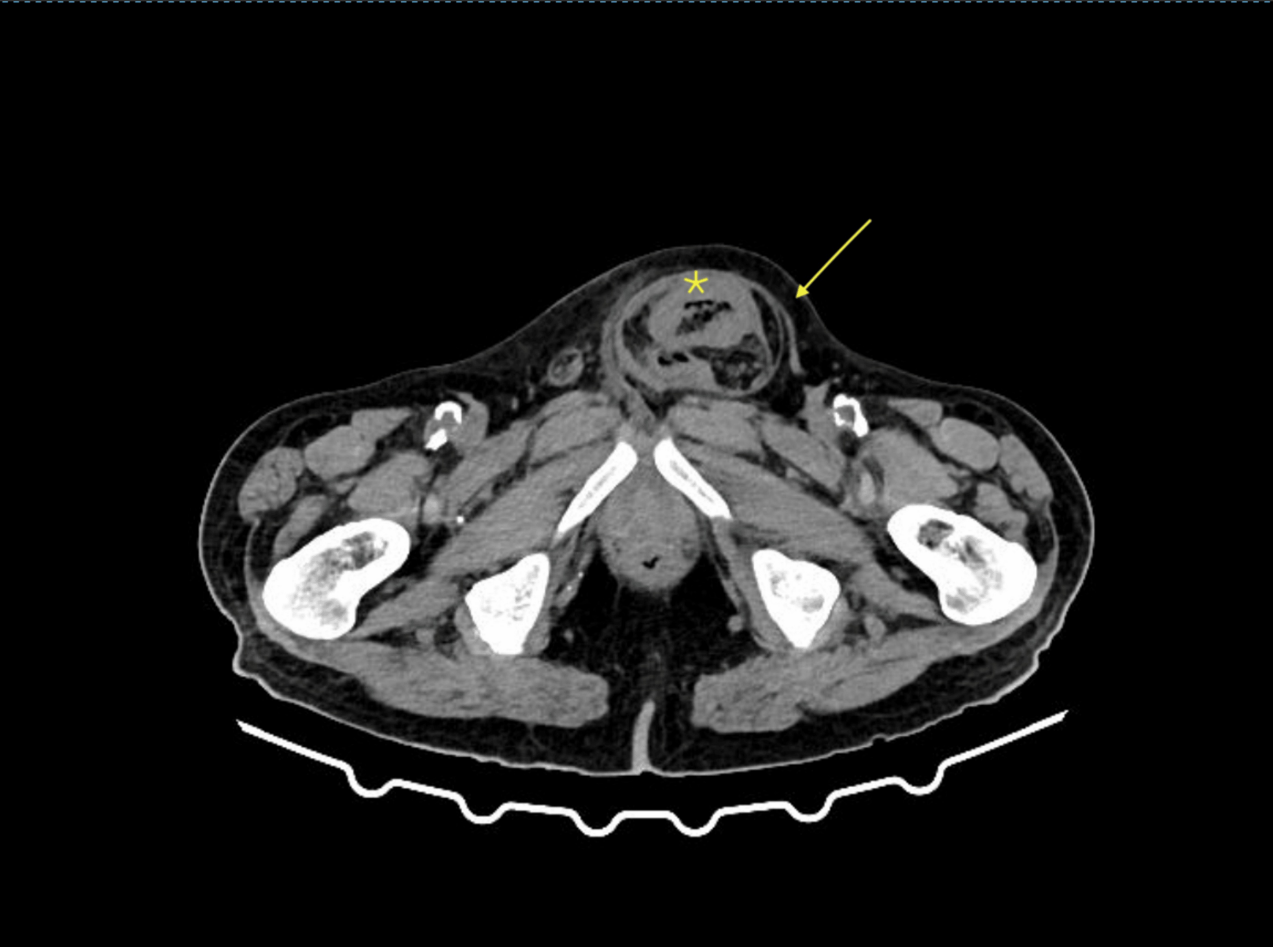
Left inguinal hernia (yellow arrow) with sigmoid colon as content. Bowel wall thickening is also evident (yellow star). The thickened bowel wall was mostly attributed to inflammation due to the incarcerated hernia but we also suspected possible malignancy concerning the age of the patient and the fact that he had never undergone colonoscopy.

Before his arrival, the patient had already undergone a computed tomography (CT) scan with oral contrast, which was ordered by his family doctor. CT showed dilated large bowel with a competent ileocecal valve (no distention of small bowel loops), while the obstruction was evident to be present in the bowel inside the inguinal hernia. Although the CT essay was not conclusive, we suspected sigmoid colon as hernia content, with significant wall thickening, which was credited to hernia incarceration (Figures 2, 3). The patient had never undergone a colonoscopy and had no known medical history concerning the bowel. Laboratory values of importance included elevated inflammation markers: WBC: 19.3 x 10^3^/μL (4-11 x 10^3^/μL), CRP: 11.89 mg/dL (<0.5 mg/dl), elevated urea: 110 mg/dL (16-40 mg/dL), and hyponatremia Na+: 128 mmol/l (136-146 mmol/l). A nasogastric tube was placed, and an antibiotic regimen of a second-generation cephalosporin along with metronidazole was initiated, along with IV fluids.

**Figure 4:**
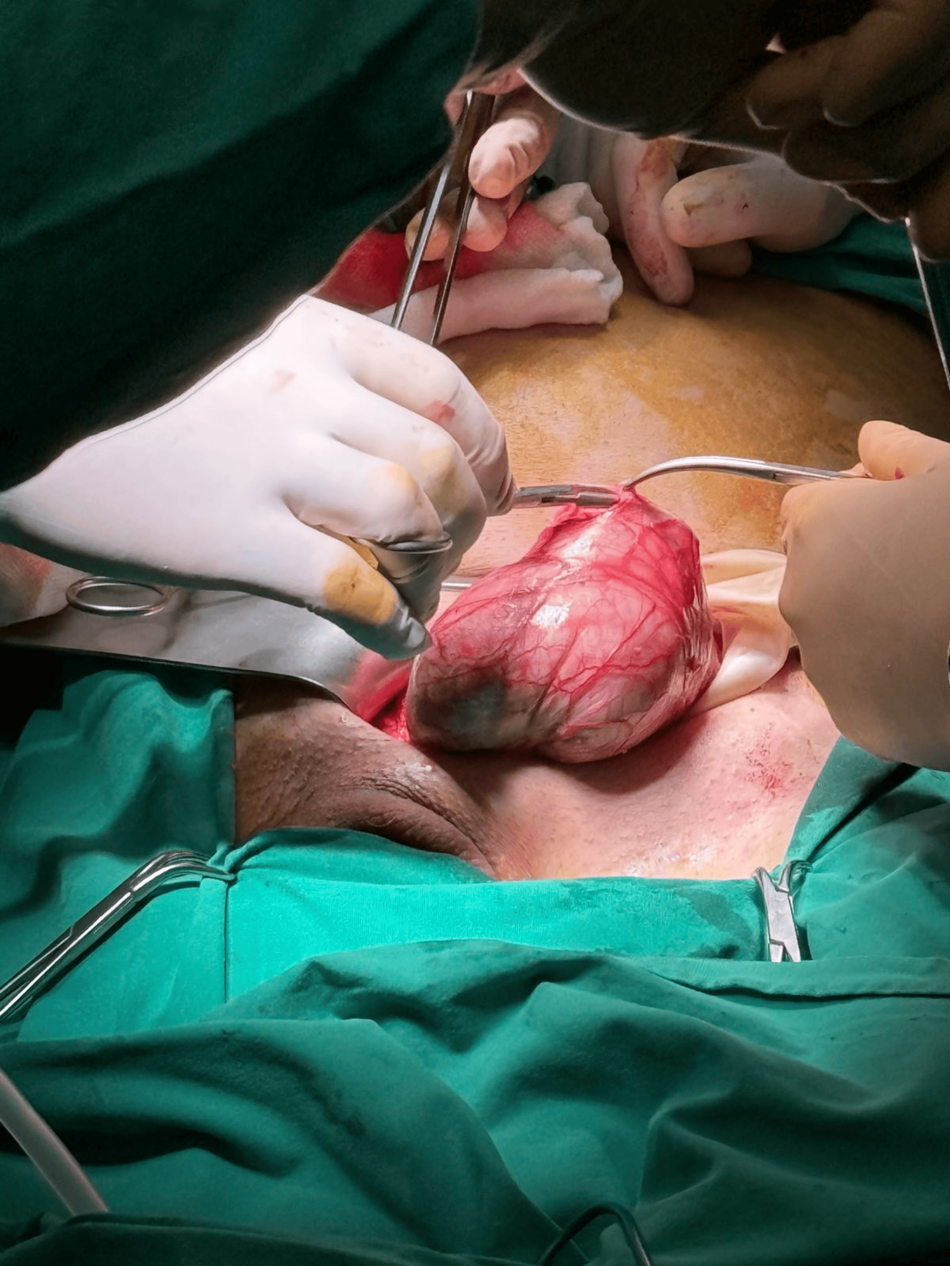
Hernia sac inspection. After dissection of the hernia sac from surrounding tissues and mobilization of the spermatic cord, we proceeded to the sac opening and inspection of the bowel and discovered the perforation site adjacent to the mass.

After initial resuscitation, the patient was then transferred to the operating room. We initially performed a left inguinal incision. The hernia sac was very firmly attached to the spermatic cord and was inflamed, containing fluid (Figure 4).

**Figure 5:**
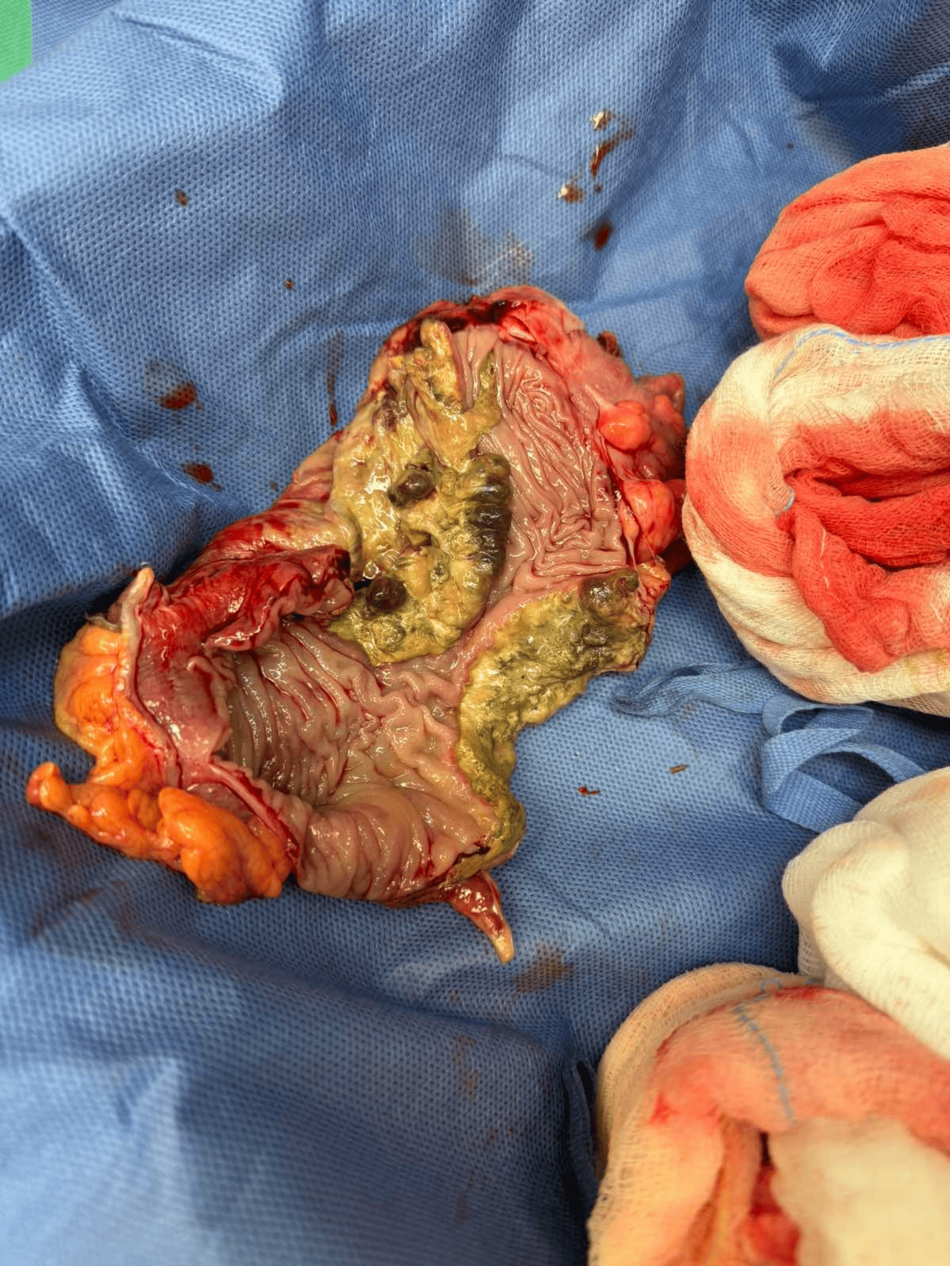
Surgical specimen. Note the tumor in the middle of the resected colon, causing an almost complete obstruction. The resected sigmoid colon, 17 cm in length, with an evident neoplasm in the middle, with at least 5 cm distance from the nearer surgical margin. Upon histological examination, the neoplasm was identified to be a sigmoid adenocarcinoma, poorly differentiated, staged as pT4aN1b according to the Union for International Cancer Control (UICC) (8th edition), with 2/26 lymph nodes positive for metastatic disease. R0 resection was achieved. No distant metastasis, peritoneal seeding, or ascites was evident inside the peritoneal cavity upon inspection or in the postoperative staging CT scan.

Upon opening the sac, we discovered the sigmoid colon and a perforation, 1 cm in diameter, of the bowel wall. While on palpation, a mass was evident near the perforated site. The surgical team opted to proceed with a midline incision to allow for adequate peritoneal cavity exploration and reduction of the sigmoid colon under direct vision. The inflamed hernia sac was tightly adhered to the left testicle and the spermatic cord, and safe tissue dissection was not possible. Considering the age and the medical history of the patient, we decided to sacrifice the left testicle, ligating the spermatic cord, to facilitate the reduction of the hernia. Hartmann’s operation was then performed utilizing a linear stapler and a vessel sealer/divider device. Because of the circumstances and the presence of peritonitis in a patient of advanced age, we did not proceed with complete mesocolic excision, although the specimen included more than an adequate number of lymph nodes (Figure 5).

**Figure 6:**
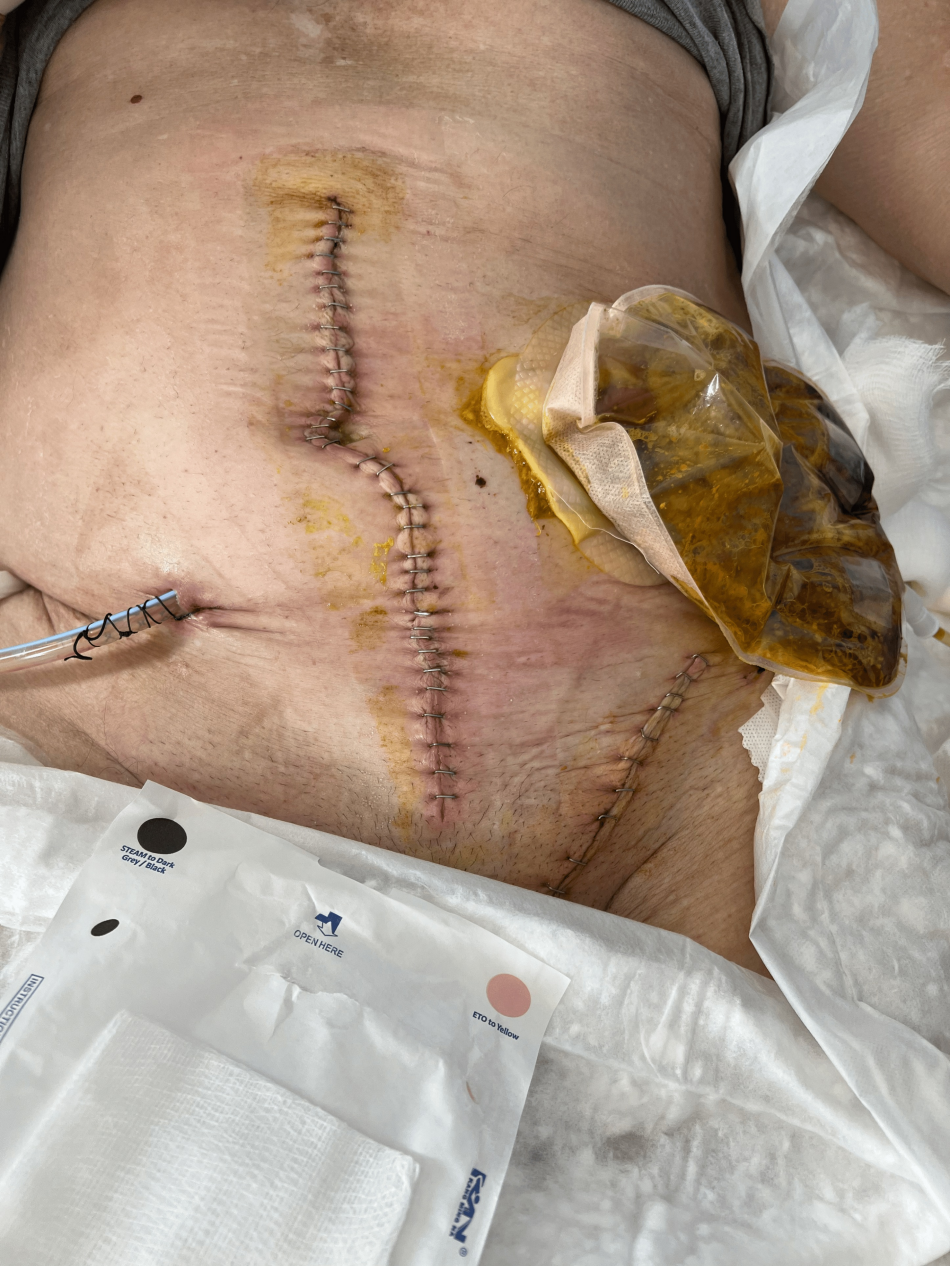
Surgical site infection at the lower part of the midline incision. Inflammation of the surgical incision is evident in its lower end. Evacuation of the fluid and thorough wash of the wound with saline was done daily .

Extensive peritoneal lavage and drain placement followed the resection. Midline wound closure was performed using a single loop PDS #2 continuous suture, while the left internal inguinal ring was closed, and a Bassini repair of the inguinal floor was utilized since we did not have sterile conditions for mesh placement. Surgical staples were used for skin closure for both incisions. The patient was transferred to the ICU and, after 24 hours, was moved to the surgical ward. The patient had a relatively uneventful recovery apart from a surgical incision infection, which was successfully treated by evacuating the fluid and washing the wound with saline (Figure 6).

The nasogastric tube was removed on the second postoperative day, and the patient was able to exit the hospital after six days. Regular follow-up visits revealed good wound closure within 14 days, while nutrition and general condition were improved. In a follow-up visit after three months, the patient remains metastasis-free. 

## Discussion

Bowel obstruction is a common cause of acute abdomen and requires surgical intervention. While large bowel obstruction is less frequent than small bowel obstruction (25% vs. 75%) [1], care must be taken, especially in elderly individuals, in whom large bowel obstruction is even more common [3]. The main etiology for colon obstruction is cancer in about 60% of the cases [4], volvulus or diverticular disease account for 30% [5], and the remaining causes are rarer, such as hernias, inflammatory bowel disease, stenosis, and endometriosis. During the last 100 years, a large shift in large bowel obstruction primary etiology has been observed, with an increasing incidence in colon cancer, reported as high as 80% in some studies [6]. The most common site of appearance for colorectal carcinoma appears to be distal to the splenic flexure (75%), with the primary location being the sigmoid colon [4]. Large bowel obstruction represents 80% of colorectal cancer emergencies, while the remaining 20% is perforation. Large bowel obstruction can present suddenly, with acute colic-like abdominal pain, abdominal distention, bloating, absence of bowel movement, and flatus, while vomiting is less frequent than in small bowel obstruction dependent on the competence of the ileocecal valve, or sub-acutely, with gradual development of symptoms, changes in bowel habits and recurrent left lower quadrant abdominal pain [7].

In a series of 150 consecutive patients suffering from acute mechanical bowel obstruction, 24% presented with large bowel obstruction. Absence of passage of flatus (90%) and/or faeces (80.6%) and abdominal distension (65.3%) were the most common symptoms and physical signs, while abdominal examination can reveal tenderness, guarding, abdominal distension, and hyperactive or absent bowel sounds [7]. Medical history reporting the presence of blood in the feces or loss of blood from the rectum may be associated with colorectal cancer. The treatment of choice depends on the cause of the large bowel obstruction. Patients with obstructive colorectal cancer should be individually approached depending on the case. Resection and primary anastomosis after peritoneal lavage seems to be an equally good, if not better, treatment compared to Hartmann’s procedure in fit patients [8]. However, we opted to perform the latter, which requires less operating time since our patient was older, large bowel obstruction was complicated by perforation, and he had serious comorbidities [9].

To our knowledge, this is one of the few reported cases presenting sigmoid adenocarcinoma as content of an incarcerated inguinal hernia [10-12]. Most authors of such reported cases also performed Hartmann’s procedure in the presence of peritonitis [12]. Inguinal hernia repair requires a non-contaminated wound, while sigmoid colon cancer excision is a clean-contaminated operation or even a contaminated operation when there is a perforated malignant tumor. In principle, a patch should not be used in such procedures for hernia repair. Simple suture repair and reconstruction of the inguinal floor is possible, such as Bassini or Halsted methods, which should be selected individually for each patient to avoid the occurrence of postoperative surgical site infection [13-14]. In a recent systematic review of the last 30 years, conducted by El Shamarka et al., 40 patients (23 in an emergency setting) had colon cancer as content of an inguinal hernia, out of which, 95.1% of the patients were males and 65.9% were over the age of 70 years, while 78% of the hernial sacs contained sigmoid colon neoplasms. Concurrent primary hernia repair and colectomy were performed in 33 patients (80.5%), while diversion stoma was done in 43.9% of the patients [15]. A narrative review published in 2022 by Zhang et al. included 16 patients with colon cancer as inguinal hernia content [16]. In all these cases, the patients were men aged between 44 and 88 years (median, 71.5 years), and most presented with left inguinal hernias (75.0%) [16]. Over 80% of these patients had inguinal hernias that contained sigmoid colon cancer (82.5%), while the others contained the descending colon and the ascending colon, and in most cases, the contents were incarcerated (82.5%) [16]. The majority of the patients underwent open surgery (82.2%), and in many, combined (abdominal and inguinal) incisions were made (62.5%), while for the colon, the Hartmann procedure was the procedure of choice (56.3%) because of the high perforation rates, and for inguinal hernia repair, primary closure (58.3%) was carried out [16].

## Conclusions

The simultaneous presence of colorectal cancer and inguinal hernia is uncommon, and detailed preoperative physical examination and imaging studies are essential to the establishment of a correct diagnosis. The selection of an appropriate surgical procedure, considering many factors, ensures the best possible therapeutic results and should be individualized for each patient. In most published cases, the described surgery was performed in an emergency setting, or carcinomas were found by surprise, resulting in suboptimal oncological treatment. With our case report, we intend to raise surgeons’ suspicion about the possible presence of malignant colon neoplasm as a content of an incarcerated inguinal hernia in order to achieve the best possible level of care.
